# Residency Program Characteristics Associated With Osteopathic Resident Representation

**DOI:** 10.7759/cureus.82442

**Published:** 2025-04-17

**Authors:** Dhimitri A Nikolla, Vatsala Sachdeva, Aviya Distefano, Avery Bryan, Vishnu Mudrakola, Melody L Milliron, Kaitlin M Bowers

**Affiliations:** 1 Department of Internal Medicine/Emergency Medicine, Lake Erie College of Osteopathic Medicine, Erie, USA; 2 Department of Emergency Medicine, Allegheny Health Network, Erie, USA; 3 Department of Medical Education, Jerry M. Wallace School of Osteopathic Medicine, Campbell University, Lillington, USA; 4 Undergraduate Studies, East Carolina University, Greenville, USA; 5 Department of Emergency Medicine, Summa Health, Akron, USA; 6 Department of Emergency Medicine, Jerry M. Wallace School of Osteopathic Medicine, Campbell University, Lillington, USA

**Keywords:** academic medical centers, community medicine, internship and residency, medical education, medical faculty, medical specialities, medical students, osteopathic medicine, osteopathic physicians

## Abstract

Objective

Osteopathic (DO) medical students encounter unique challenges applying and matriculating to postgraduate training programs. To better understand these challenges and where DO students matriculate for residency, we aimed to examine program characteristics associated with higher DO resident representation among postgraduate programs in the United States.

Methodology

We conducted a retrospective, cross-sectional study of Accreditation Council for Graduate Medical Education (ACGME) accredited residency programs within the 10 largest specialties (i.e., with the most training spots) cataloged within the Fellowship and Residency Electronic Interactive Database Access (FREIDA) dataset in 2022. We explored program-level characteristics associated with DO representation, DOs constituting ≥33% of residents, presenting descriptive statistics and risk ratios (RR) with 95% confidence intervals (CIs) clustered at the sponsor level. Each level of non-binary categorical variables was analyzed as an indicator variable, while continuous variables were rescaled by dividing by two standard deviations to facilitate comparing the strength of associations between DO representation and each program-level characteristic.

Results

Of the 3,364 programs from the 10 specialties included in the study, 2,284 (67.9%) had <33% DO residents, and 1,080 (32.1%) had ≥33%. Former American Osteopathic Association (AOA) accreditation was more common at programs with ≥33% DO residents (*n *= 461, 42.7%) than programs with <33% DO residents (*n *= 108, 4.7%) (RR 3.65, 95% CI 3.29-4.06, *P* < 0.001). Other variables associated with greater DO representation included osteopathic recognition, community setting, and family medicine specialty. A U.S. Medical Licensing Examination (USMLE) completion requirement for DO applicants was less common at programs with ≥33% DO residents (*n* = 38, 3.5%) than programs with <33% DO residents (*n *= 738, 32.3%) (RR 0.12, 95% CI 0.09-0.17, *P* < 0.001). Other variables associated with lower DO representation included university setting, more programs at the same sponsoring institution, full-time faculty, first-year positions, U.S. allopathic medical school graduates (USMD) residents, and sponsorship of J1, H1B, and F1 visas.

Conclusions

Postgraduate training programs in the United States with ≥33% DO residents were more common in community settings with fewer USMD residents, fewer first-year positions, fewer programs at the same sponsoring institution, and fewer full-time faculty as well as among programs with former AOA accreditation, osteopathic recognition, and no USMLE requirement for DO applicants.

## Introduction

Most resident physicians enrolled in postgraduate medical training programs in the United States are graduates of U.S. allopathic medical schools (USMD), U.S. osteopathic medical schools (DO), or international medical schools (IMG) [1]. Currently, all U.S. postgraduate programs are accredited by the Accreditation Council for Graduate Medical Education (ACGME) [2]. However, this single accreditation occurred only recently in 2020, following a five-year transition period during which programs accredited by the American Osteopathic Association (AOA) transitioned to ACGME accreditation [2,3]. Since the implementation of single accreditation, studies examining program characteristics associated with matriculating DO residents have been limited in scope [4] and have not included post-2020 data.

Importance

Understanding where DO students matriculate for postgraduate training can inform efforts to ensure osteopathic postgraduate training opportunities to meet the physician workforce's needs. There is a projected shortage of physicians in the United States [5]. DOs help fill this demand, as 28% of U.S. medical students are from osteopathic medical schools [6]. However, DO students regularly encounter unique challenges matriculating to postgraduate programs despite a single accreditation.

Although DOs have much greater match success than IMGs, they still have lower match rates than USMDs [7], which may be due to several reasons. While DOs are eligible to complete the allopathic medical licensing examinations (i.e., United States Medical Licensing Examination, or USMLE), the USMLE is not required for DOs to obtain medical licensure. Nevertheless, many postgraduate programs require DO applicants to complete the USMLE to get an interview, despite completing their required osteopathic medical licensing boards (i.e., Comprehensive Osteopathic Medical Licensing Examination of the United States, or COMLEX-USA) [8]. Although DOs who complete the USMLE have higher COMLEX-USA scores than those who do not [9], the USMLE requirement for DOs by many programs may contribute to increased match success by DOs who complete the USMLE [10]. Additionally, even programs that do not require USMLE completion for DO applicants may misinterpret COMLEX-USA scores [11,12].

Nevertheless, DOs still have lower match success than USMDs among cohorts with similar USMLE scores [7]. This illustrates that board examination scores do not entirely determine match success and highlights deficiencies DO applicants have compared to USMDs, including reduced research output [13-15], limited sub-internship opportunities due to home institutions without affiliated postgraduate programs [16-18], and ineligibility for or barriers to obtaining away rotations [19,20]. The impact of these variables may result in differences in program characteristics between those with and without DO representation.

Goals of the investigation

DO students face many challenges pursuing postgraduate medical training after a single accreditation. Ensuring postgraduate training opportunities for DO students requires a better understanding of where DO students matriculate for residency. Therefore, we aimed to examine postgraduate program characteristics associated with higher DO resident representation among ACGME-accredited programs in the United States.

## Materials and methods

Study design and setting

We conducted a retrospective, cross-sectional study of ACGME-accredited residency programs cataloged within the Fellowship and Residency Electronic Interactive Database Access (FREIDA) dataset from the American Medical Association (AMA) [21]. According to the Allegheny Health Network Institutional Review Board, which reviewed our study protocol, the study did not meet the definition for human subjects research as defined by the federal code of regulations: 45 CFR 46.102(f).

Data collection

Data contained within FREIDA are reported from each program collected via an annual survey (i.e., National Graduate Medical Education Census) distributed by the AMA and the Association of American Medical Colleges (AAMC). We obtained the data from the AMA through a data licensing agreement in April 2023. Data were collected at the program level and analyzed at the program level. Data were last updated between June 2022 and December 2022 for all programs in the dataset.

Programs

Specialties were limited to the 10 with the most training spots in 2022, as programs in smaller, more specialized fields matriculate fewer DOs. Furthermore, program characteristics associated with the matriculation of DOs to programs from these smaller, more competitive specialties may not be the same as more popular specialties. Therefore, we included all programs contained within FREIDA from anesthesiology, emergency medicine, family medicine, internal medicine, neurology, obstetrics and gynecology, orthopedic surgery, pediatrics, psychiatry, and general surgery. This constitutes 3,364 of 5,308 (63.4%) programs participating in the 2022 National Resident Matching Program (NRMP) match [1].

Variables

Our primary outcome was programs with ≥33% DO residents. We selected this outcome because about a third of U.S. medical students are from osteopathic schools [6]. And, describing where DOs are distributed could inform efforts to expand graduate-level osteopathic training opportunities [22] and address imbalances [23]. We derived this dichotomized outcome from a FREIDA variable containing the three-year average percentage of DO residents at each program. We selected independent variables that might reflect institutional resources or culture, program size, admission practices, and DO friendliness. Variables reflecting institutional resources or culture included program type (i.e., university, community, community-based university-affiliated, military, other), number of programs at the same sponsoring institution, meal allowance, onsite childcare, subsidized childcare, housing stipend, first-year salary, proportion of faculty that are female, and maximum days of paid sick and vacation leave. The number of programs at the same sponsoring institution was calculated as the number of programs at the sponsor of the observed program and was obtained by linking sponsors' identification numbers to programs’ identification numbers from the ACGME website [24]. Variables reflecting program size included the number of first-year positions, interviews offered, and full-time faculty. Variables reflecting admission practices included the proportions of USMD and IMG residents (3-year average), the proportion of female residents (three-year average), visa sponsorship (J1, H1B, F1), and a reported minimum board examination score for an interview offer (either USMLE Step 1 or 2, or COMLEX-USA Level 1 or 2). Variables reflecting DO friendliness included former AOA accreditation, osteopathic recognition, and a USMLE requirement for DO applicants to obtain an interview (either USMLE Step 1 or 2) [25]. The former AOA program variable was obtained from the AOA website and linked to the FREIDA data by program identification numbers [3]. Finally, we also examined specialty given its known association with DO resident matriculation [7].

Statistical analysis

We provide descriptive statistics of each independent variable stratified by our outcome, <33% vs. ≥33% DO residents. Values for the primary outcome were missing for 246 (7.3%) programs; therefore, we imputed the missing values using multiple imputation. We considered a strategy using the proportion of IMG and USMD residents to impute missing DO resident proportions, but the missing data were correlated between the variables. Therefore, we used a random forest model to perform nonparametric multiple imputation, imputing missing primary outcome values predicted from the following variables: specialty, program type, number of first-year positions, number of interviews offered, reported minimum board examination score, USMLE requirement for DO applicants, osteopathic recognition, and former AOA accreditation (out-of-bag error 0.484 and proportion of falsely classified 0.072).

We report means with standard deviations (SD), medians with interquartile range (IQR), and counts with percentages for each variable stratified by DO representation (i.e., <33% vs. ≥33% DO residents). Normality was assessed visually with histograms and quantile-quantile plots. To better understand the strength of the association between our primary outcome and each independent variable, we rescaled all continuous variables. By dividing each continuous variable value by two standard deviations of the variable, all variables, continuous or binary, would be on similar scales [26]. We then calculated risk ratios (RR) with 95% confidence intervals (CI), clustered at the sponsor level [27], using relative risk regression with univariate log-binomial models [28]. Since former AOA accreditation is strongly associated with factors that encourage DO matriculation, like not requiring USMLE completion for DO applicants [8], we repeated the above analyses after excluding programs with former AOA accreditation [3]. Finally, to better understand the distribution of programs regarding representation of DOs compared to USMDs and IMGs, we plotted a stacked bar chart of program counts by representation of each physician type, ranging from 0% to 100%.

Our analysis was intended to be descriptive and exploratory. Since we used an existing dataset, we did not perform an a priori sample size or power calculation [29]. Each level of non-binary categorical variables was analyzed as a separate indicator variable. To account for the multiplicity of the 37 comparisons made, we indicate which associations have *P*-values below the Bonferroni-corrected threshold (*P* < 0.0014). All analyses and visualizations were performed using R (version 4.4.2, R Foundation for Statistical Computing, Vienna, Austria), with the logbin package used for relative risk regression [28].

## Results

**Figure 1:**
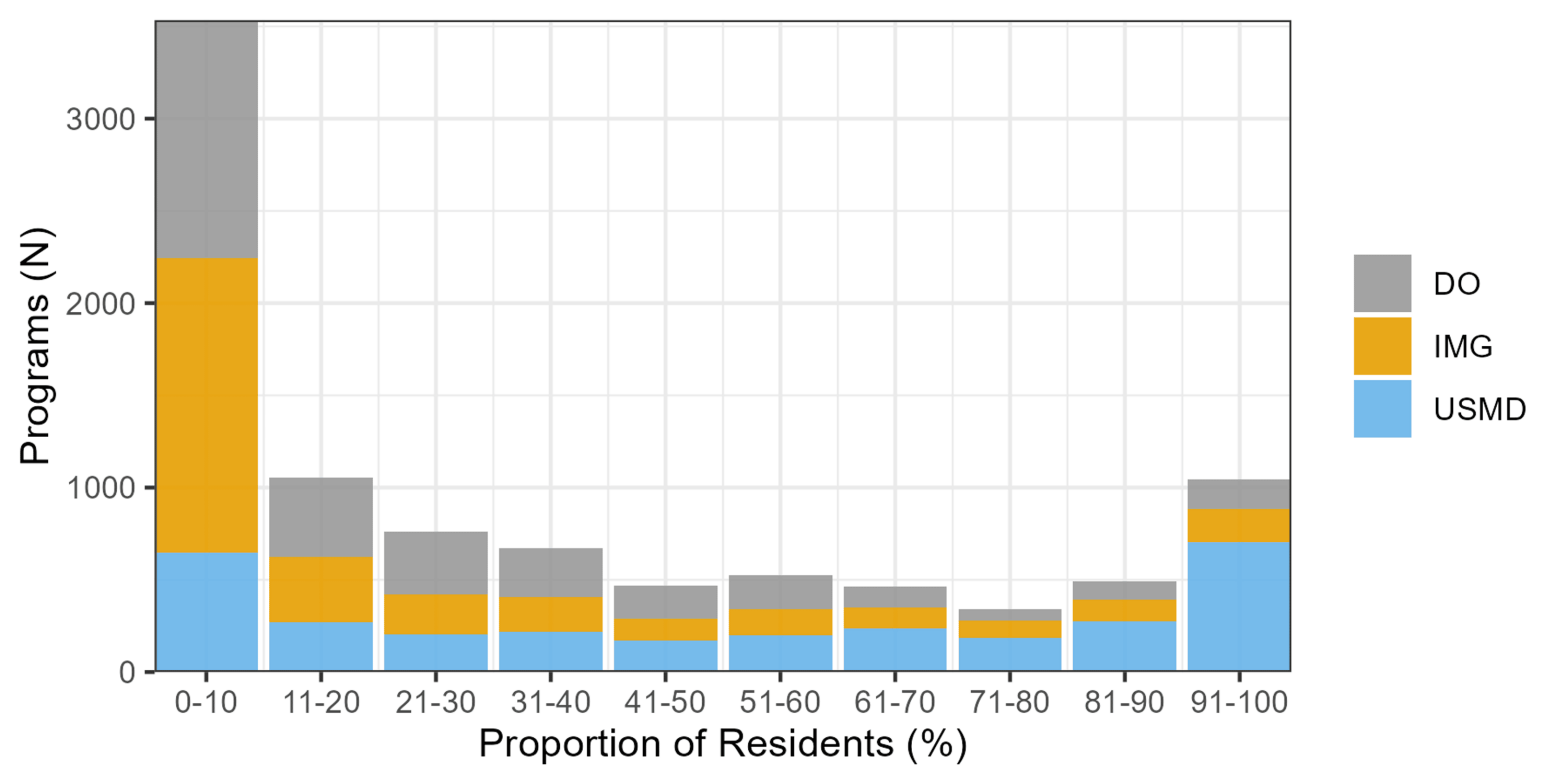
Stacked bar chart of programs by the proportion of each resident type. The plot displays a stacked bar chart of programs by the proportion of each resident type (i.e., USMD, DO, IMG) (*n* = 3,118 with complete data). Program counts are not mutually exclusive between resident types since each program will have a proportion of USMDs, DOs, and IMGs, even if a proportion is zero. DO, osteopathic medical school graduate; IMG, international medical school graduate; USMD, allopathic medical school graduate

Of the 3,364 programs from the 10 specialties included in the study, 2284 (67.9%) had <33% DO residents, and 1080 (32.1%) had ≥33%. Most programs have low proportions of DOs, and few programs have high proportions of DOs (Figure 1). The percentage of USMD residents was higher at programs with < vs. ≥ 33% DO residents, median 75.0% (IQR 30.4 to 93.6) vs. 20.0% (5.3 to 40.2). Former AOA accreditation was less common at programs with < vs. ≥ 33% DO residents, 108 (4.7%) vs. 461 (42.7%) programs. Osteopathic recognition was also less common at programs with < vs. ≥ 33% DO residents, 52 (2.3%) vs. 167 (15.5%) programs. A USMLE requirement for DOs was more common at programs with < vs. ≥ 33% DO residents, 738 (32.3%) vs. 38 (3.5%) programs. The number of programs at the same sponsoring institution was notably higher at programs with < vs. ≥ 33% DO residents, a median of 38.0 (IQR 11.0 to 82.0) vs. 13.0 (IQR 5.0 to 34.0) programs (Table 1).

**Table 1:** Program characteristics associated with DO resident representation. AOA, American Osteopathic Association; CI, confidence interval; DO, osteopathic medical school graduate; IMG, international medical school graduate; IQR, interquartile range; SD, standard deviation; USMD, allopathic medical school graduate; USMLE, United States Medical Licensing Examination

Variable	<33% DO residents	≥33% DO residents
Total, *N*	2284	1080
Program type, *n* (%)		
Community-based university-affiliated hospital	889 (38.9)	531 (49.2)
Community hospital	335 (14.7)	405 (37.5)
Military-based	42 (1.8)	28 (2.6)
Other	14 (0.6)	3 (0.3)
University hospital	1,004 (44.0)	113 (10.5)
Specialty, *n* (%)		
Anesthesiology	108 (4.7)	56 (5.2)
Emergency medicine	165 (7.2)	118 (10.9)
Family medicine	392 (17.2)	351 (32.5)
Internal medicine	434 (19.0)	183 (16.9)
Neurology	138 (6.0)	39 (3.6)
Obstetrics and gynecology	221 (9.7)	77 (7.1)
Orthopedic surgery	169 (7.4)	39 (3.6)
Pediatrics	166 (7.3)	50 (4.6)
Psychiatry	221 (9.7)	81 (7.5)
General surgery	270 (11.8)	86 (8.0)
Former AOA, *n* (%)	108 (4.7)	461 (42.7)
Minimum board examination score, *n* (%)	1,646 (72.1)	739 (68.4)
USMLE required for DOs, *n* (%)		
Yes	738 (32.3)	38 (3.5)
Missing	102 (4.5)	62 (5.7)
J1 visa sponsored, *n* (%)		
Yes	1,729 (75.7)	491 (45.5)
Missing	57 (2.5)	51 (4.7)
H1B visa sponsored, *n* (%)		
Yes	518 (22.7)	111 (10.3)
Missing	91 (4.0)	62 (5.7)
F1 visa sponsored, *n* (%)		
Yes	351 (15.4)	78 (7.2)
Missing	105 (4.6)	63 (5.8)
Meal allowance, *n* (%)		
Yes	2,064 (90.4)	916 (84.8)
Missing	22 (1.0)	29 (2.7)
Osteopathic recognition, *n* (%)		
Yes	52 (2.3)	167 (15.5)
Missing	84 (3.7)	288 (26.7)
Onsite child care, *n* (%)		
Yes	552 (24.2)	183 (16.9)
Missing	22 (1.0)	30 (2.8)
Subsidized childcare, *n* (%)		
Yes	188 (8.2)	55 (5.1)
Missing	22 (1.0)	30 (2.8)
Housing stipend, *n* (%)		
Yes	309 (13.5)	95 (8.8)
Missing	22 (1.0)	30 (2.8)
First-year positions, median (IQR)	8.0 (6.0-14.0)	6.0 (4.0-9.0)
Missing	2 (0.1)	0 (0.0)
Number of interviews, median (IQR)	101.0 (72.0-160.0)	86.0 (50.0-120.0)
Missing, *n* (%)	53 (2.3)	27 (2.5)
Percent DO residents, median (IQR)	5.9 (0.0-16.7)	56.1 (42.9-81.0)
Missing, *n* (%)	138 (6.0)	108 (10.0)
Percent IMG residents, median (IQR)	9.5 (0.0-54.1)	8.3 (0.0-23.8)
Missing, *n* (%)	138 (6.0)	108 (10.0)
Percent USMD residents, median (IQR)	75.0 (30.4-93.6)	20.0 (5.3-40.2)
Missing, *n* (%)	138 (6.0)	108 (10.0)
Percentage of female residents, median (IQR)	48.4 (36.7-62.8)	45.8 (33.3-60.0)
Missing, *n* (%)	138 (6.0)	108 (10.0)
Percent female faculty, mean (SD)	41.0 (20.1)	41.0 (21.9)
Missing, *n* (%)	161 (7.0)	191 (17.7)
First-year salary, median (IQR)	60,442 (56,831-64,040)	58,958 (55,869-61,959)
Missing, *n* (%)	97 (4.2)	78 (7.2)
Maximum paid days of medical leave, median (IQR)	30.0 (20.0-44.5)	21.0 (20.0-40.0)
Missing, *n* (%)	965 (42.3)	481 (44.5)
Maximum paid days of vacation, median (IQR)	20.0 (15.0-21.0)	20.0 (15.0-20.0)
Missing, *n* (%)	94 (4.1)	86 (8.0)
Maximum paid days of sick leave, median (IQR)	12.0 (8.0-14.0)	10.0 (5.0-12.0)
Missing, *n* (%)	747 (32.7)	534 (49.4)
Number of full-time faculty, median (IQR)	23.0 (8.0-49.0)	8.0 (3.0-18.2)
Programs per sponsor, median (IQR)	38.0 (11.0-82.0)	13.0 (5.0-34.0)
Missing, *n* (%)	8 (0.4)	5 (0.5)

**Figure 2:**
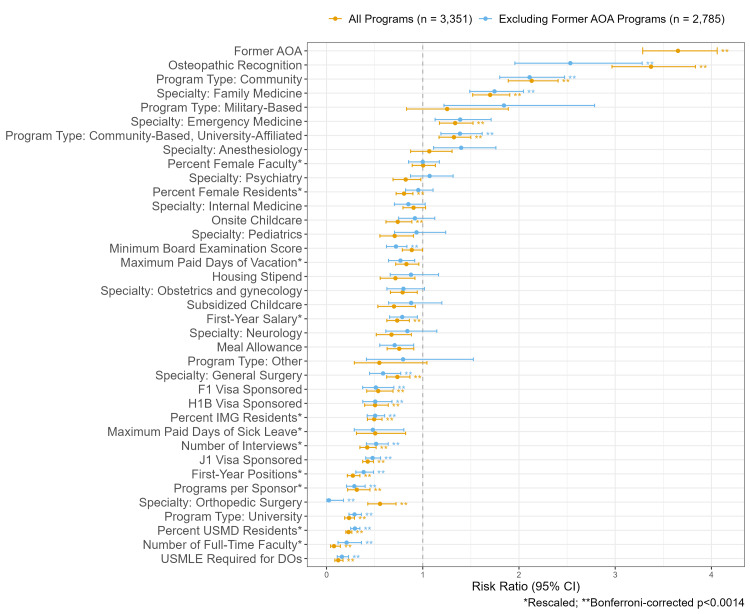
Plot of risk ratios for ≥33% DO residents. The plot displays univariate risk ratios for associations between independent variables and ≥33% DO residents among all programs and those without former AOA accreditation. Continuous variables were rescaled by dividing each value by two standard deviations of the variable. Confidence intervals are clustered at the sponsor level; therefore, 13 programs were excluded for missing sponsor numbers. AOA, American Osteopathic Association; CI, confidence interval; DO, osteopathic medical school graduate; IMG, international medical school graduate; RR, risk ratio; USMD, allopathic medical school graduate; USMLE, United States Medical Licensing Examination

Former AOA accreditation had the strongest positive association with ≥33% DO residents (RR 3.65, 95% CI 3.29-4.06), while the number of full-time faculty had the strongest negative association with ≥33% DO residents (RR 0.08, 95% CI 0.04-0.14) (Figure 2, Table 2). The results were similar after excluding former AOA programs (Tables 2-3, Figure 2). However, after excluding former AOA programs, osteopathic recognition had the strongest positive association with ≥33% DO residents (RR 2.53, 95% CI 1.96-3.28), while orthopedic surgery specialty had the strongest negative association with ≥33% DO residents (RR 0.02, 95% CI 0.00-0.18) (Figure 2, Table 2).

**Table 2:** Risk ratios for ≥33% DO residents. *Rescaled by dividing each value by 2 standard deviations of the variable. **P-value is significant after Bonferroni correction for 37 comparisons (*P* < 0.0014). AOA, American Osteopathic Association; CI, confidence interval; DO, osteopathic medical school graduate; IMG, international medical school graduate; NA, not applicable; RR, risk ratio; USMD, allopathic medical school graduate; USMLE, United States Medical Licensing Examination

Variable	All programs		Excluding former AOA	
	RR (95% CI)	P-value	RR (95% CI)	P-value
Former AOA	3.65 (3.29-4.06)	<0.001**	NA	NA
Osteopathic Recognition	3.37 (2.97-3.83)	<0.001**	2.53 (1.96-3.28)	<0.001**
Program Type: Community	2.13 (1.89-2.41)	<0.001**	2.11 (1.80-2.47)	<0.001**
Specialty: Family Medicine	1.70 (1.52-1.90)	<0.001**	1.75 (1.49-2.05)	<0.001**
Specialty: Emergency Medicine	1.34 (1.17-1.52)	<0.001**	1.39 (1.13-1.71)	0.002
Program Type: Community-Based, University-Affiliated	1.32 (1.17-1.50)	<0.001**	1.39 (1.19-1.62)	<0.001**
Program Type: Military-Based	1.25 (0.83-1.89)	0.280	1.84 (1.22-2.79)	0.004
Specialty: Anesthesiology	1.07 (0.87-1.30)	0.520	1.40 (1.11-1.76)	0.004
Percent Female Faculty*	1.00 (0.89-1.13)	0.953	1.00 (0.85-1.17)	1.000
Specialty: Internal Medicine	0.90 (0.79-1.03)	0.128	0.85 (0.71-1.03)	0.089
Minimum Board Examination Score	0.89 (0.79-1.00)	0.043	0.72 (0.62-0.84)	<0.001**
Maximum Paid Days of Vacation*	0.83 (0.72-0.96)	0.012	0.77 (0.64-0.92)	0.003
Specialty: Psychiatry	0.82 (0.69-0.98)	0.029	1.07 (0.87-1.32)	0.509
Percent Female Residents*	0.81 (0.72-0.90)	<0.001**	0.95 (0.82-1.11)	0.531
Specialty: Obstetrics and Gynecology	0.79 (0.66-0.94)	0.009	0.80 (0.63-1.02)	0.069
Meal Allowance	0.76 (0.63-0.91)	0.003	0.71 (0.55-0.91)	0.006
Onsite Childcare	0.74 (0.62-0.89)	0.001**	0.92 (0.75-1.12)	0.410
Specialty: General Surgery	0.74 (0.63-0.87)	<0.001**	0.59 (0.45-0.77)	<0.001**
First-Year Salary*	0.73 (0.63-0.86)	<0.001**	0.79 (0.66-0.94)	0.010
Housing Stipend	0.72 (0.56-0.92)	0.009	0.88 (0.66-1.16)	0.365
Specialty: Pediatrics	0.71 (0.55-0.90)	0.006	0.94 (0.71-1.24)	0.640
Subsidized Childcare	0.70 (0.53-0.92)	0.011	0.88 (0.64-1.20)	0.415
Specialty: Neurology	0.68 (0.52-0.88)	0.004	0.84 (0.62-1.15)	0.271
Specialty: Orthopedic Surgery	0.56 (0.43-0.72)	<0.001**	0.02 (0.00-0.18)	<0.001**
Program Type: Other	0.55 (0.29-1.04)	0.067	0.80 (0.41-1.53)	0.491
F1 Visa Sponsored	0.54 (0.42-0.69)	<0.001**	0.51 (0.38-0.70)	<0.001**
Maximum Paid Days of Sick Leave*	0.51 (0.31-0.82)	0.006	0.48 (0.29-0.80)	0.005
H1B Visa Sponsored	0.50 (0.40-0.64)	<0.001**	0.51 (0.38-0.68)	<0.001**
Percent IMG Residents*	0.49 (0.42-0.58)	<0.001**	0.50 (0.42-0.60)	<0.001**
J1 Visa Sponsored	0.43 (0.38-0.49)	<0.001**	0.48 (0.41-0.56)	<0.001**
Number of Interviews*	0.42 (0.35-0.52)	<0.001**	0.52 (0.41-0.64)	<0.001**
Programs per Sponsor*	0.31 (0.22-0.45)	<0.001**	0.29 (0.21-0.40)	<0.001**
First-Year Positions*	0.27 (0.22-0.35)	<0.001**	0.39 (0.30-0.49)	<0.001**
Program Type: University	0.23 (0.19-0.29)	<0.001**	0.29 (0.23-0.36)	<0.001**
Percent USMD Residents*	0.23 (0.20-0.26)	<0.001**	0.29 (0.25-0.35)	<0.001**
USMLE Required for DOs	0.12 (0.09-0.17)	<0.001**	0.16 (0.11-0.23)	<0.001**
Number of Full-Time Faculty*	0.08 (0.04-0.14)	<0.001**	0.21 (0.12-0.36)	<0.001**

**Table 3:** Residency program characteristics associated with DO resident representation, excluding former AOA programs. AOA, American Osteopathic Association; CI, confidence interval; DO, osteopathic medical school graduate; IMG, international medical school graduate; IQR, interquartile range; SD, standard deviation; USMD, allopathic medical school graduate; USMLE, United States Medical Licensing Examination

Variable	<33% DO residents	≥33% DO residents
Total, *N*	2,176	619
Program type, *n* (%)		
Community-based, university-affiliated hospital	827 (38.0)	299 (48.3)
Community hospital	298 (13.7)	192 (31.0)
Military-based	42 (1.9)	28 (4.5)
Other	14 (0.6)	3 (0.5)
University hospital	995 (45.7)	97 (15.7)
Specialty, *n* (%)		
Anesthesiology	108 (5.0)	47 (7.6)
Emergency medicine	160 (7.4)	68 (11.0)
Family medicine	348 (16.0)	178 (28.8)
Internal medicine	398 (18.3)	97 (15.7)
Neurology	134 (6.2)	31 (5.0)
Obstetrics and gynecology	221 (10.2)	48 (7.8)
Orthopedic surgery	169 (7.8)	1 (0.2)
Pediatrics	156 (7.2)	41 (6.6)
Psychiatry	214 (9.8)	66 (10.7)
General surgery	268 (12.3)	42 (6.8)
Minimum board examination score, *n* (%)	1,555 (71.5)	384 (62.0)
USMLE required for DOs, *n* (%)		
Yes	734 (33.7)	35 (5.7)
Missing	97 (4.5)	45 (7.3)
J1 visa sponsored, *n* (%)		
Yes	1,655 (76.1)	331 (53.5)
Missing	55 (2.5)	38 (6.1)
H1B visa sponsored, *N* (%)		
Yes	504 (23.2)	70 (11.3)
Missing	89 (4.1)	45 (7.3)
F1 visa sponsored, *n* (%)		
Yes	339 (15.6)	46 (7.4)
Missing	102 (4.7)	45 (7.3)
Meal allowance, *n* (%)		
Yes	1,972 (90.6)	515 (83.2)
Missing	22 (1.0)	29 (4.7)
Osteopathic recognition, *n* (%)		
Yes	23 (1.1)	27 (4.4)
Missing	33 (1.5)	18 (2.9)
Onsite child care, *n* (%)		
Yes	531 (24.4)	134 (21.6)
Missing	22 (1.0)	30 (4.8)
Subsidized childcare, *n* (%)		
Yes	179 (8.2)	42 (6.8)
Missing	22 (1.0)	30 (4.8)
Housing stipend, *n* (%)		
Yes	303 (13.9)	72 (11.6)
Missing	22 (1.0)	30 (4.8)
First-year positions, median (IQR)	8.0 (6.0-14.0)	8.0 (5.0-10.0)
Missing	2 (0.1)	0 (0.0)
Number of interviews, median (IQR)	100.0 (70.0-160.0)	96.0 (60.0-130.0)
Missing, *n* (%)	51 (2.3)	18 (2.9)
Percent DO Residents, median (IQR)	5.3 (0.0-16.7)	45.9 (37.9-57.1)
Missing, *n* (%)	137 (6.3)	100 (16.2)
Percentage of IMG residents, median (IQR)	8.3 (0.0-48.0)	10.0 (0.0-22.9)
Missing, *n* (%)	137 (6.3)	100 (16.2)
Percentage of USMD residents, median (IQR)	77.8 (34.8-94.3)	36.8 (20.8-50.0)
Missing, *n* (%)	137 (6.3)	100 (16.2)
Percentage of female residents, median (IQR)	48.1 (36.4-62.9)	47.4 (34.2-62.5)
Missing, *n* (%)	137 (6.3)	100 (16.2)
Percent Female Faculty, mean (SD)	40.9 (20.2)	43.0 (19.9)
Missing, *n* (%)	148 (6.8)	88 (14.2)
First-Year Salary, median (IQR)	60,537 (56,904 to 64,101)	59,625 (56,613 to 62,400)
Missing, *n* (%)	93 (4.3)	52 (8.4)
Maximum Paid Days of Vacation, median (IQR)	20.0 (15.0 to 21.0)	20.0 (15.0 to 20.0)
Missing, *n* (%)	89 (4.1)	61 (9.9)
Maximum Paid Days of Sick Leave, median (IQR)	12.0 (9.0 to 14.0)	10.0 (5.0 to 12.0)
Missing, *n* (%)	701 (32.2)	249 (40.2)
Number of Full-Time Faculty, median (IQR)	24.0 (8.0 to 50.0)	11.0 (5.0 to 27.0)
Programs Per Sponsor, median (IQR)	39.0 (11.0-83.0)	16.0 (5.0-36.0)
Missing, *n* (%)	8 (0.4)	2 (0.3)

## Discussion

Postgraduate programs with <33% vs. ≥33% DO residents differed across many characteristics, including factors related to institutional resources or culture, program size, admission practices, and DO friendliness. The associations between DO representation and markers of DO friendliness, like a USMLE requirement for DO applicants, were expected but offer some additional evidence that DOs matriculate to programs that support their training or at least do not exclude them. Even after excluding former AOA programs, the association between a USMLE requirement for DOs and ≥33% DO residents remained, suggesting that the association is not entirely driven by a history of training DOs (Figure 2, Table 2). Furthermore, this association is consistent with a prior study examining COMLEX-USA acceptance among emergency medicine programs [8].

While markers of DO friendliness have a probable mechanism to affect DO representation, some associations we observed may be more representative of the consequences of matriculation pressures on DOs. For example, USMD resident representation is associated with lower DO representation (Figure 2, Table 2). This is also illustrated by the large number of programs with low DO representation compared to USMD representation, which has a more uniform distribution (Figure 1). Therefore, DOs and IMGs are likely filling spots that are not taken by USMDs. An example of this is the 2024 emergency medicine match, where DO and IMG applicants to emergency medicine programs markedly increased compared to a drop in USMD applicants [30]. Given the associations we observed, programs matriculating DOs are often at community, non-university hospitals with fewer programs at the sponsoring institution, fewer residents (i.e., fewer first-year positions), and fewer full-time faculty (Figure 2, Table 2). However, as a result, DO residents more commonly matriculate to programs with lower salaries (i.e., lower first-year salaries) and worse benefits (i.e., fewer paid vacation and sick days as well as less access to meal allowances and onsite childcare) (Table 2). 

These results inform efforts to expand postgraduate osteopathic training opportunities in several ways. First, while DOs do compete with IMGs for spots, i.e., IMG representation is negatively associated with DO representation (Table 2), DOs still have markedly better match success than IMGs [7]. Therefore, it is most pressing for DOs to better compete with USMDs, who generally have better match success [7]. While efforts to deter programs from excluding DOs may help [11,23], DO students cannot control these exclusionary practices. Therefore, DO students may increase postgraduate training opportunities by acquiring credentials and experiences that make them more competitive, including taking the USMLE [10], conducting research [15], and completing sub-internships or away rotations at postgraduate training sites [18]. In particular, this strategy will likely increase access to competitive specialties, like orthopedic surgery, where DOs are markedly underrepresented (Tables 1-2) [13]. For example, only one of 170 (0.6%) orthopedic surgery programs without former AOA accreditation has ≥ 33% DO residents (Table 3). Osteopathic medical schools and other stakeholders may improve access to postgraduate training opportunities for all DO students by increasing opportunities to acquire experiences that make them more competitive (e.g., research gap year, sub-internships at postgraduate training sites). Lastly, more competitive applicants will likely not only take postgraduate training spots in more competitive specialties but also at institutions with better resources and benefits. Osteopathic medical schools and other stakeholders should work towards ensuring parity in benefits (e.g., salary, availability of onsite childcare, paid vacation, and sick days) for all residents.

Limitations

Our analysis was exploratory and not intended to draw causal conclusions. Furthermore, the direction of associations is unclear from these cross-sectional data (e.g., DO applicants pursuing certain specialties vs. programs from certain specialties pursuing DO applicants). However, some associations may be important even if they are confounded (e.g., the associations between DO representation and lower first-year salary). Therefore, we did not attempt any multivariable analyses to adjust for confounders. The cross-sectional nature of the study also makes the temporality unknown for certain associations. For example, we do not know if a USMLE requirement for DO applicants was in place at the time each applicant applied. We only know that the USMLE requirement was in place at the time of data collection. Our primary outcome had a small, but not insignificant, degree of missingness. We used nonparametric multiple imputation of the missing outcome values to combat bias from missingness of the outcome variable, but this also assumes the missing values are missing at random and can be predicted by observed variables [31,32]. Similarly, some independent variables had high missingness, like paid vacation days (1,281 of 3,364, or 38.1%, missing), which may bias our results. Since 2022, when the data were collected, USMLE Step 1 and COMLEX-USA Level 1 have transitioned to pass-fail grading [33], which makes the generalizability of certain variables, like minimum board examination scores and USMLE requirements for DOs, more uncertain. We cannot confirm the accuracy of the reported data. The data comes directly from programs via a survey, but their responses may not reflect practice. For example, admission practices, like requiring the USMLE for DOs, may change with varying applicant popularity and selectivity of the program over time, especially if programs cannot fill positions, e.g., the drop in applications for emergency medicine after the COVID-19 pandemic [34]. Our study cannot inform DO representation from specialties not included in the analysis.

## Conclusions

Among the 10 specialties with the most training spots in 2022, postgraduate training programs with ≥33% DO residents were more common at programs with fewer USMD residents from community settings with fewer first-year positions, less programs at the sponsoring institution, and fewer full-time faculty as well as among programs with former AOA accreditation, osteopathic recognition, and no USMLE requirement for DO applicants to obtain an interview. Programs with ≥33% DO residents also had worse benefits, including lower first-year salaries, fewer paid vacation and sick days, and less access to meal allowances and onsite childcare. These results highlight matriculation pressures for DO students transitioning to postgraduate training and inform efforts to expand postgraduate training opportunities for DO students.
